# Epidemiology of malaria resistance to Artemisinin: resistance or temporary tolerance

**DOI:** 10.1186/1475-2875-9-S2-I5

**Published:** 2010-10-20

**Authors:** Carol Hopkins Sibley

**Affiliations:** 1 Centre for Tropical Medicine, University of Oxford, Oxford, OX3 7LJ, UK

## 

Artemisinin-based combination therapies (ACTs) are the recommended first-line treatment for *Plasmodium falciparum* malaria. While efficacy remains high in many areas of the world, prolonged parasite clearance times following treatment with some ACTs and seven-day artemisinin therapy have been observed on the Thai/Cambodian border [1].

Despite increasing investment in control, we still do not know the extent to which artemisinin resistant falciparum malaria has spread. Such knowledge is vital to plan an effective public health response to either eliminate or control the disease within the region, and to guard against spread to Africa where 85% of global deaths from malaria are recorded. The potentially catastrophic consequence of failing to contain these genetically altered, resistant parasites is clear [2].

The WorldWide Antimalarial Resistance Network (WWARN) has built a web-based informatics platform enabling the malaria community to collate, analyse and share information on different aspects of antimalarial efficacy. WWARN has also provided a platform for researchers to share information on artemisinin resistance with the goal of identifying molecular markers of artemisinin resistance.

Dr Sibley will review the current understanding of the epidemiology of artemisinin resistance, identify the organizational gaps in that information, and discuss how scientists can contribute to the project.
